# Dietary Patterns and the Risk of Prediabetes in Taiwan: A Cross-Sectional Study

**DOI:** 10.3390/nu12113322

**Published:** 2020-10-29

**Authors:** Yi-Cheng Hou, Han-Chih Feng, I-Shiang Tzeng, Chan-Yen Kuo, Ching-Feng Cheng, Jing Hui Wu, Shwu-Huey Yang

**Affiliations:** 1 Department of Nutrition, Taipei Tzu Chi Hospital, Buddhist Tzu Chi Medical Foundation, New Taipei City 231405, Taiwan; d507103002@tmu.edu.tw (Y.-C.H.); sandt7796@gmail.com (H.-C.F.); arita.wu@gmail.com (J.H.W.); 2 School of Nutrition and Health Sciences, Taipei Medical University, Taipei City 110301, Taiwan; 3 Department of Research, Taipei Tzu Chi Hospital, Buddhist Tzu Chi Medical Foundation, New Taipei City 231405, Taiwan; istzeng@gmail.com (I.-S.T.); cykuo863135@gmail.com (C.-Y.K.); 4 Department of Pediatrics, Taipei Tzu Chi Hospital, Buddhist Tzu Chi Medical Foundation, New Taipei City 231405, Taiwan; chengcf@mail.tcu.edu.tw; 5 Institute of Biomedical Sciences, Academia Sinica, Taipei City 115201, Taiwan; 6 Department of Pediatrics, Tzu Chi University, Hualien County 970374, Taiwan; 7 Nutrition Research Center, Taipei Medical University Hospital, Taipei city 110301, Taiwan; 8 Research Center of Geriatric Nutrition, College of Nutrition, Taipei Medical University, Taipei city 110301, Taiwan

**Keywords:** dietary pattern, prediabetes, factor analysis, fasting glucose, glycated hemoglobin, Taiwan, cross-sectional study

## Abstract

Background: prediabetes prevention and management are the main methods used to combat the prevalence of diabetes. Exploratory factor analysis is an upcoming method that is successful in identifying dietary patterns that correlate with healthy or unhealthy outcomes. Aim: this study aims to identify dietary patterns in Taiwan that are associated with the risk of prediabetes. Methods: anthropometric, blood glucose, 3 d/24 h dietary records, and food frequency questionnaire data were collected from subjects recruited at Taipei Tzu-Chi Hospital. The following five dietary patterns were identified using factor analysis: Western, prudent, convenience, Asian traditional, and continental. This cross-sectional study compares tertiles of dietary patterns and analyzes the significance of the characteristics. Results: the Western and the prudent patterns are the major dietary patterns found in other studies. A higher factor loading in the Western pattern is significantly related to a higher risk of prediabetes. A higher factor loading in the continental pattern is significantly related to a lower risk of prediabetes. Conclusion: decreasing meat and seafood consumption while increasing egg, coffee, and milk consumption may be associated with a decreased risk for prediabetes.

## 1. Introduction

Diabetes mellitus is prevalent worldwide. The economic burden it brings to both patients and healthcare systems has been a serious issue [1]. Prediabetes is a high-risk state defined by fasting glucose levels of 100–125 mg/dL, glycated hemoglobin (HbA1c) of 5.7–6.4% or glucose tolerance test resulting in 140–199 mg/dL two hours after ingesting a standardized 75 g glucose solution [2,3]. The Nutrition and Health Survey in Taiwan 2013–2016 shows that 29.6% of adults in Taiwan have prediabetes defined by impaired fasting glucose alone [4]. Despite improvements in diabetes care in Taiwan, prevalence and expenses continue to rise [5,6]. Recent studies have shown the possibility that complications associated with diabetes may have already occurred during the prediabetic state [7,8,9], making prediabetes prevention and reversal a priority.

Prediabetic patients are recommended lifestyle modifications, such as dietary changes, exercise, and weight control [10]. Managing or preventing prediabetes via dietary intervention requires personalized design. However, various standardized interventions have already been proven effective [11,12]. In order to provide a more general guideline to the public, we used exploratory factor analysis to identify dietary patterns that correlate with the risk of prediabetes. Each dietary pattern is a group of food items that represents a type of dietary habit. Dietary habits may have originated from culture, tradition, restaurant sets, grocery availability, and lifestyle. Factor analysis had been utilized in literatures on prediabetes and diabetes [13,14,15,16]. To our knowledge, not much research in Taiwan was aimed at the risk of prediabetes using dietary pattern methods. The goal of this study was to evaluate the level of risk of major dietary patterns in terms of impaired fasting glucose and abnormal HbA1c levels. The results of this study will allow us to define dietary index scores that can be used to promote healthy dietary habits.

## 2. Materials and Methods

In this cross-sectional study, 199 subjects between 20 and 65 years of age were recruited between 2014 and 2019 at the Taipei Tzu-Chi Hospital through the Divisions of Family Medicine and Metabolism, and the Healthcare Center. Participant’s body weights and heights were measured using a weight-height scale (DS-102, Dongsahn Jenix, Seoul, Korea). Measurement was performed once by the same individual at the beginning of enrollment. The enrolled participants were instructed by the instructor in the hospital. Moreover, HbA1c, blood glucose levels, and cholesterol were assayed using an enzyme immunoassay, point-of-care testing, and spectrophotometry, respectively. The exclusion criteria included diabetes, psychiatric or neurological illness, surgery within the past 10 years, hypertension, dyslipidemia, smoking, thyroid dysfunction, asthma, adrenal dysfunction, parathyroid dysfunction, cancer, pregnancy, or renal dialysis.

Anthropometric data, 3 day/24 h dietary records, food frequency questionnaire, and blood glucose data were collected from the subjects. The 3 day/24 h dietary records provide intake data for nutrients, and were completed and then returned by the subjects. The food frequency questionnaire was validated in Chinese for use in Taiwan [17]. The blood glucose level was determined by measuring fasting glucose (mg/dL) and HbA1c (%) levels.

The results of the food frequency questionnaire were analyzed using factor analysis. Each dietary pattern corresponds to an eigenvalue. We set the threshold for the eigenvalue to be 1.6, resulting in five dietary patterns. The Varimax rotation was used to define the factor loading of food items, and was set to 0.3.

The statistical analysis considered the use of tertiles for each dietary pattern. A generalized linear model was used to determine the significance of each characteristic. The odds ratios of impaired fasting glucose and abnormal HbA1c were computed using logistic regression using one crude and two multivariate models.

## 3. Results

Items in the food frequency questionnaire, with a factor loading of at least 0.3 under an eigenvalue of at least 1.6, were considered and discussed. Table 1 shows the five dietary patterns obtained and their corresponding food items. The pattern with the largest eigenvalue, the Western pattern, contains seafood, meat, viscera, and processed products. The second pattern, the prudent pattern, contains vegetables, fruits, root or stem starch, and soy bean products. The third pattern, the convenience pattern, contains dumplings, buns, processed fruits, and nut products. The fourth pattern, the Asian traditional pattern, contains Chinese pastries, sticky rice, congee, and rice. The fifth pattern, the Continental pattern, contains eggs, coffee, milk, and snacks.

For each pattern, the subjects were grouped into tertiles based on the scores obtained from the food frequency questionnaire and the factor loadings. For the Western pattern, we found that the tertile with higher scores had significantly higher body mass index (BMI), vitamin B12, and fasting glucose, but lower simple sugar and folic acid, as shown in Table 2. For the prudent pattern, we found that the tertile with higher scores had significantly higher age, carbohydrate, and vitamin C, but lower cholesterol, as shown in Table 3. For the convenience pattern, we found that the tertile with higher scores had significantly higher age and fasting glucose, but lower cholesterol and eicosapentaenoic acid (EPA), as shown in Table 4. For the Asian traditional pattern, we found no characteristics significantly related to the tertiles, as shown in Table 5. For the Continental pattern, we found that the tertile with higher scores had significantly higher simple sugar, vitamin E, and folic acid, but lower age, fasting glucose, and HbA1c, as shown in Table 6.

Based on the definition of prediabetes with fasting glucose, Table 7 shows the odds ratio based on the first lowest tertile using three models: a crude model, a multivariate model controlling age and BMI, and a multivariate model controlling age, BMI, and total energy intake. In the crude model, the second and third tertiles of the Western pattern had a 311% and a 361% chance of prediabetes, respectively. The third tertile of the convenience pattern had a 240% chance of prediabetes. The third tertile of the Continental pattern had a 36% chance of prediabetes. In the age- and BMI-controlled models, the second and third tertiles of the Western pattern had a 305% and a 339% chance of prediabetes, respectively. The third tertile of the Continental pattern had a 44% chance of prediabetes. In the last multivariate model, the second and third tertiles of the Western pattern had a 295% and a 330% chance of prediabetes, respectively. Lastly, the third tertile of the Continental pattern had a 42% chance of prediabetes. The other tertiles either did not have a significant relationship or had a 100% relative risk in the 95% confidence interval.

Based on the definition of prediabetes with respect to HbA1c, Table 8 shows the odds ratio between tertiles using three models: a crude model, a multivariate model controlling age and BMI, and a multivariate model controlling age, BMI, and total energy intake. In the crude model, the third tertile of the Continental pattern had a 29% chance of prediabetes. In the age- and BMI-controlled models, the third tertile of the Continental pattern had a 38% chance of prediabetes. In the last multivariate model, the third tertile of the Continental pattern had a 37% chance of prediabetes. The other tertiles either did not have a significant relationship or had a 100% relative risk in the 95% confidence interval.

## 4. Discussion

The global threat of diabetes brought about an abundance of literatures that attempt to determine the best methods for intervention [18]. Diet has always been the forefront factor in discussions. Single nutrients, food items, food groups, specific diets, diet scores, and dietary patterns are all being studied to better understand the effectiveness of various dietary interventions [11,12,16,19,20,21]. Literature about prediabetes have extended research on diabetes to a broader and an earlier stage [2,22,23]. On one hand, methodologies were focused on preventing prediabetic patients from progressing into diabetes [24]. On the other hand, preventing prediabetes became a priority due to irreversible harm occurring during the prediabetic state [2,7,10,25,26]. Therefore, this study aimed to identify dietary patterns in Taiwan that are significantly related to the risk of prediabetes. The goal is to raise awareness for unhealthy dietary habits and to promote healthy diets.

The Western and the prudent patterns were named after the Western and the prudent diets, respectively [27,28]. The Western pattern, which is significantly related to BMI and fasting glucose, matched our expectations. A higher consumption of the Western pattern items increased the risks of obesity and prediabetes. Moreover, a lower simple sugar and folic acid intake could be a sign of a less balanced dietary habit. Comparing the bottom tertile, the middle and top tertiles of the Western pattern have over triple the risk of prediabetes in all models. It referred to a dietary pattern with a high level of protein and fat. Being a relatively unhealthy diet, it was not able to balance blood sugar. Moreover, Western diets may be associated with type 2 diabetes through mechanisms beyond glucose balance [28].

The prudent pattern contained food items that were considered important in maintaining a healthy balanced diet. The relationship between consumption of the prudent pattern items and age indicated that older adults were more health conscious. The prudent pattern was a balanced diet. A balanced diet consisted of 50% to 60% total calories (dietary fiber involved in sugar), 10–20% protein, and 20–30% fat. Individuals had a higher score in the prudent pattern as percentage nutrient consumption was relatively high. The carbohydrates in the prudent pattern included root starchy vegetables (complex sugars, dietary fiber), vegetables (dietary fiber), seed products (complex sugars), and peanut rice milk (complex sugars). Most compounds were complex carbohydrates and dietary fiber, which belonged to a low-GI (glycemic index) diet. However, these had a limited impact on blood sugar [29]. A lower cholesterol and a higher vitamin C intake both matched the design of the prudent diet. There was no significant relationship between the prudent pattern and the risk of prediabetes. This was unexpected and indicated that the lowest and the highest tertiles were not that far apart in terms of prudent pattern item consumption. Furthermore, this pattern referred to a healthy diet with many fruits and vegetables. A previous study indicated that there was no association between this diet and type 2 diabetes or fasting blood glucose [28].

The convenience pattern was named based on the common features of the food items. Buns, dumplings, processed fruit, and nut products were common choices for a convenient and a quick meal. A higher consumption was related to older subjects, a lower intake of cholesterol and EPA, and a higher fasting glucose. The increased risk of prediabetes was no longer significant in multivariate models, which meant that the age difference across the tertiles was partly the reason for a higher risk. This diet was considered favorable and practical for the elderly, as the items listed may be eaten alone.

The traditional Asian pattern consisted of food items common in Taiwan, but rare outside of Asia. Items were all staple foods. We found no significant relationship between traditional Asian pattern consumption and other characteristics, and should be the result of most subjects consuming staple foods in similar amounts. Asians are accustomed to porridge and rice as principal food. However, these lacked seasonings, vegetables, and protein. Moreover, these belong to complex carbohydrates, and individuals may also choose low-GI types, such as grain porridge (or rice), brown rice, or oatmeal rice [30]. They eat rice to increase blood sugar, severed with vegetables, and proteins that stabilize blood sugar [31].

The continental pattern was represented by eggs and coffee. Our findings matched literature regarding egg and coffee consumption [32,33]. A higher consumption was significantly related to younger subjects, a higher simple sugar, vitamin E, and folic acid intake, and a lower risk of prediabetes. When comparing the top tertile to the bottom tertile, the risk of prediabetes was reduced to almost one-third. The multivariate model showed that the lower risk of prediabetes was partially due to the younger subjects. However, the multivariate models showed that the risk was reduced to 40%. Moreover, this pattern corresponded to an eigenvalue just above our threshold. However, this was considered to be the most significant pattern for reducing the risk of prediabetes. The continental pattern was closely related to the Western breakfast pattern, which usually consisted of an egg, a cup of flavored sugary coffee, a muffin, and a cup of milk served with lettuce salad. The Continental breakfast was more popular among younger age groups as compared to the Convenience pattern. The coffee-type beverage was a flavored milk beverage rich in simple sugars and protein. The relationship between lettuce salad, vitamin E, and folic acid were relatively high. Moreover, blood sugar remained unaffected by egg consumption [32]. Milk contained carbohydrates and proteins, but no simple sugar. Furthermore, increasing blood sugar and HbA1c levels when used with lettuce salad were found to be difficult [31]. Additionally, consuming black coffee instead of flavored coffee was also found to not affect blood sugar [33].

The strength of this study mainly revolved around the localized food frequency questionnaire, which allowed our resulting dietary patterns to match closely with daily dietary habits. This was the first study to use dietary patterns in identifying the risk of prediabetes in Taiwan. Previous literature on dietary patterns chose prediabetic patients as subjects [15]. Our main limitation revolved around the cross-sectional design of the study and the number of subjects, as Neyman Bias may have ensued. Exploratory factor analysis was used to identify major factors representing the subjects. A larger number of participants would have improved the accuracy in representing the society. Different laboratory methods may have caused measurement bias. Confounding factors only included the age and the BMI of the participants. To our records, a small proportion of prediabetes subjects already know their glucose status. We recommend a longer period of study to provide more stable results about dietary changes among individuals over time.

## 5. Conclusions

Our study concluded that having a lower consumption of meat, viscera, and seafood, while having a higher consumption of eggs, coffee, and milk may be associated with significant reductions in the risk of prediabetes in Taiwan.

## Figures and Tables

**Table 1:** Dietary patterns based on factor loading from food frequency questionnaire.

	Western	Prudent	Convenience	Asian Traditional	Continental
Deep sea fish	0.724				
Meat	0.670				
Viscera	0.667				
Seafood	0.483	0.347			
Processed meat	0.434		0.326		
Fried pancakes	0.379				
Flavoring vegetables	0.330				
Vegetables		0.811			
Root or stem starch		0.571			
Soy bean products		0.560			
Fresh fruits		0.323		−0.313	
Seed products		0.304			
Rice milk		0.302			
Savory buns			0.652		
Dumplings			0.615		
Processed fruits			0.606		
Nuts or nut products			0.574		
Breakfast grains			0.529		
Chinese pastries				0.686	
Sticky rice				0.529	
Sticky rice sweets				0.492	
Congee				0.479	
Rice				0.415	
Eggs					0.719
Coffee beverages					0.687
Snacks		0.424			0.476
Milk					0.317

**Table 2:** Distribution of characteristics by the tertiles of the Western pattern.

	Western			*p* Trend ^2^
	T1 ^1^ (*n* = 66)	T2 ^1^ (*n* = 67)	T3 ^1^ (*n* = 66)	
Age (year)	51.82 ± 9.02	51.15 ± 10.86	51.91 ± 9.33	0.957
BMI (kg/m^2^)	23.01 ± 3.11	24.27 ± 3.43	24.45 ± 3.34	0.013
Energy (kcal)	1488.36 ± 370.08	1559.25 ± 558.33	1558.76 ± 459.16	0.390
Protein (g)	53.98 ± 20.05	62.07 ± 28.69	57.55 ± 18.98	0.829
Fat (g)	58.81 ± 44.43	71.34 ± 60.42	77.79 ± 80.04	0.087
Carbohydrate (g)	255.44 ± 112.63	266.67 ± 216.31	298.60 ± 283.39	0.252
Fiber (g)	24.60 ± 176.90	3.73 ± 3.65	3.59 ± 3.37	0.238
Cholesterol (g)	255.90 ± 200.55	292.81 ± 181.24	257.11 ± 156.82	0.969
Simple sugar (g)	14.10 ± 32.93	8.85 ± 14.61	4.26 ± 7.61	0.008
Iron (mg)	11.04 ± 8.77	11.90 ± 14.86	9.55 ± 4.64	0.411
Zinc (mg)	7.55 ± 2.72	7.80 ± 2.59	33.15 ± 209.70	0.225
Vitamin E (mg)	6.53 ± 4.32	8.30 ± 16.54	5.58 ± 5.14	0.599
Vitamin B12 (μg)	2.91 ± 4.66	3.45 ± 2.81	4.26 ± 3.72	0.042
Folic acid (μg)	177.19 ± 241.37	108.71 ± 176.99	83.26 ± 117.36	0.004
Vitamin C (mg)	117.44 ± 81.94	135.38 ± 97.97	105.64 ± 91.24	0.456
EPA (mg)	62.73 ± 100.56	58.63 ± 91.01	58.98 ± 146.22	0.852
DHA (mg)	182.02 ± 534.20	127.50 ± 372.88	201.56 ± 839.54	0.855
Fasting glucose (mg/dL)	94.61 ± 12.91	100.94 ± 18.62	101.26 ± 14.87	0.016
HbA1c (%)	5.54 ± 0.48	5.68 ± 0.68	5.72 ± 0.55	0.069

^1^ Tertiles of dietary pattern scores; ^2^ *p* trend was calculated using generalized linear models for continuous variables. BMI, body mass index. EPA, eicosapentaenoic acid. DHA, docosahexaenoic acid. HbA1c, glycated hemoglobin.

**Table 3:** Distribution of characteristics by the tertiles of the prudent pattern.

	Prudent			*p* Trend ^2^
	T1 ^1^ (*n* = 66)	T2 ^1^ (*n* = 67)	T3 ^1^ (*n* = 66)	
Age (year)	49.24 ± 10.54	51.75 ± 9.11	53.88 ± 9.07	0.006
BMI (kg/m^2^)	24.21 ± 3.24	23.86 ± 3.23	23.67 ± 3.57	0.364
Energy (kcal)	1613.44 ± 540.13	1534.89 ± 445.62	1458.41 ± 402.64	0.058
Protein (g)	60.48 ± 23.02	56.68 ± 18.89	56.45 ± 26.96	0.534
Fat (g)	67.70 ± 43.34	75.45 ± 87.44	64.73 ± 50.71	0.789
Carbohydrate (g)	233.57 ± 130.69	278.71 ± 205.30	308.24 ± 280.67	0.047
Fiber (g)	2.32 ± 1.99	3.50 ± 2.91	26.11 ± 176.74	0.181
Cholesterol (g)	316.27 ± 213.13	263.13 ± 159.67	226.86 ± 153.78	0.004
Simple sugar (g)	9.15 ± 13.26	7.47 ± 14.16	10.62 ± 31.99	0.696
Iron (mg)	9.90 ± 5.91	9.44 ± 3.79	13.17 ± 16.36	0.069
Zinc (mg)	7.72 ± 2.88	32.78 ± 208.13	7.62 ± 2.50	0.996
Vitamin E (mg)	6.75 ± 5.15	5.77 ± 6.38	7.93 ± 16.02	0.513
Vitamin B12 (μg)	3.85 ± 4.12	3.04 ± 2.78	3.74 ± 4.39	0.875
Folic acid (μg)	141.35 ± 255.51	109.60 ± 135.71	118.19 ± 155.25	0.483
Vitamin C (mg)	93.16 ± 75.60	126.32 ± 84.01	139.12 ± 105.92	0.004
EPA (mg)	72.55 ± 110.32	65.18 ± 134.77	42.52 ± 93.82	0.133
DHA (mg)	205.05 ± 561.56	186.37 ± 827.46	118.77 ± 345.89	0.419
Fasting glucose (mg/dL)	97.30 ± 13.73	100.10 ± 18.84	99.41 ± 14.67	0.448
HbA1c (%)	5.55 ± 0.63	5.65 ± 0.54	5.74 ± 0.55	0.067

^1^ Tertiles of dietary pattern scores; ^2^ *p* trend was calculated using generalized linear models for continuous variables. Abbreviations: BMI, body mass index. EPA, eicosapentaenoic acid. DHA, docosahexaenoic acid. HbA1c, glycated hemoglobin.

**Table 4:** Distribution of characteristics by the tertiles of the convenience pattern.

	Convenience			*p* Trend ^2^
	T1 ^1^ (*n* = 66)	T2 ^1^ (*n* = 67)	T3 ^1^ (*n* = 66)	
Age (year)	49.24 ± 10.56	50.67 ± 9.94	54.97 ± 7.66	0.001
BMI (kg/m^2^)	23.91 ± 3.08	24.21 ± 3.59	23.61 ± 3.35	0.614
Energy (kcal)	1529.97 ± 614.33	1544.10 ± 383.11	1532.54 ± 375.70	0.975
Protein (g)	55.04 ± 24.87	60.71 ± 25.85	57.86 ± 17.76	0.373
Fat (g)	79.60 ± 83.54	68.22 ± 59.83	60.16 ± 38.32	0.079
Carbohydrate (g)	316.84 ± 292.05	250.38 ± 152.93	253.74 ± 172.14	0.093
Fiber (g)	2.40 ± 2.45	3.64 ± 3.80	25.89 ± 176.75	0.187
Cholesterol (g)	292.22 ± 212.94	289.21 ± 166.11	224.45 ± 150.74	0.030
Simple sugar (g)	6.22 ± 8.87	8.42 ± 12.72	12.58 ± 33.82	0.090
Iron (mg)	9.03 ± 4.56	12.06 ± 15.29	11.39 ± 7.88	0.189
Zinc (mg)	7.39 ± 2.83	33.08 ± 208.09	7.64 ± 2.51	0.991
Vitamin E (mg)	5.60 ± 4.49	6.44 ± 6.99	8.40 ± 15.90	0.121
Vitamin B12 (μg)	3.05 ± 4.01	3.93 ± 3.65	3.63 ± 3.80	0.379
Folic acid (μg)	156.96 ± 258.13	112.76 ± 126.64	99.37 ± 154.52	0.080
Vitamin C (mg)	105.97 ± 98.74	121.06 ± 91.23	131.64 ± 81.88	0.106
EPA (mg)	73.19 ± 101.92	78.95 ± 145.95	27.89 ± 79.39	0.022
DHA (mg)	164.53 ± 389.06	280.78 ± 936.14	63.45 ± 262.67	0.340
Fasting glucose (mg/dL)	96.00 ± 11.74	98.30 ± 19.89	102.55 ± 14.37	0.018
HbA1c (%)	5.57 ± 0.41	5.63 ± 0.73	5.74 ± 0.55	0.092

^1^ Tertiles of dietary pattern scores; ^2^ *p* trend was calculated using generalized linear models for continuous variables. Abbreviations: BMI, body mass index. EPA, eicosapentaenoic acid. DHA, docosahexaenoic acid. HbA1c, glycated hemoglobin.

**Table 5:** Distribution of characteristics by the tertiles of the Asian traditional pattern.

	Asian Traditional			*p* Trend ^2^
	T1 ^1^ (*n* = 66)	T2 ^1^ (*n* = 68)	T3 ^1^ (*n* = 65)	
Age (year)	51.80 ± 10.58	50.93 ± 8.92	52.17 ± 9.77	0.830
BMI (kg/m^2^)	23.65 ± 3.47	23.70 ± 3.11	24.39 ± 3.44	0.206
Energy (kcal)	1460.35 ± 413.22	1580.85 ± 547.82	1564.61 ± 426.22	0.203
Protein (g)	56.16 ± 19.81	59.12 ± 21.04	58.32 ± 28.00	0.750
Fat (g)	71.38 ± 63.21	72.77 ± 79.80	63.63 ± 41.15	0.487
Carbohydrate (g)	278.77 ± 257.44	284.85 ± 228.60	256.39 ± 145.80	0.554
Fiber (g)	3.69 ± 4.36	3.16 ± 2.18	25.42 ± 178.20	0.224
Cholesterol (g)	286.41 ± 200.45	261.82 ± 163.10	258.00 ± 177.39	0.370
Simple sugar (g)	10.61 ± 15.22	7.19 ± 13.03	9.48 ± 31.79	0.765
Iron (mg)	10.49 ± 5.61	10.53 ± 7.77	11.50 ± 15.32	0.579
Zinc (mg)	7.59 ± 2.63	7.81 ± 2.53	33.49 ± 211.32	0.221
Vitamin E (mg)	8.05 ± 15.84	6.40 ± 5.52	5.98 ± 6.53	0.257
Vitamin B12 (μg)	3.47 ± 4.59	3.97 ± 3.94	3.17 ± 2.68	0.653
Folic acid (μg)	130.24 ± 125.63	140.53 ± 269.22	97.24 ± 131.80	0.319
Vitamin C (mg)	137.62 ± 104.89	113.10 ± 74.24	108.00 ± 90.43	0.063
EPA (mg)	65.81 ± 101.66	56.73 ± 103.92	57.86 ± 136.68	0.693
DHA (mg)	169.85 ± 471.02	150.93 ± 459.20	190.55 ± 839.78	0.847
Fasting glucose (mg/dL)	97.97 ± 20.29	100.35 ± 14.32	98.46 ± 12.00	0.860
HbA1c (%)	5.62 ± 0.69	5.68 ± 0.55	5.64 ± 0.48	0.830

^1^ Tertiles of dietary pattern scores; ^2^ *p* trend was calculated using generalized linear models for continuous variables. Abbreviations: BMI, body mass index. EPA, eicosapentaenoic acid. DHA, docosahexaenoic acid. HbA1c, glycated hemoglobin.

**Table 6:** Distribution of characteristics by the tertiles of the Continental pattern.

	Continental			*p* Trend ^2^
	T1 ^1^ (*n* = 67)	T2 ^1^ (*n* = 66)	T3 ^1^ (*n* = 66)	
Age (year)	54.52 ± 8.30	51.44 ± 9.96	48.86 ± 10.15	0.001
BMI (kg/m^2^)	23.58 ± 3.06	24.82 ± 3.57	23.35 ± 3.24	0.692
Energy (kcal)	1475.21 ± 360.19	1550.48 ± 557.64	1581.95 ± 467.80	0.190
Protein (g)	54.28 ± 17.87	58.37 ± 21.63	61.02 ± 28.46	0.239
Fat (g)	64.00 ± 65.13	64.74 ± 42.26	79.30 ± 77.66	0.166
Carbohydrate (g)	250.91 ± 181.67	281.80 ± 242.04	288.23 ± 220.50	0.320
Fiber (g)	4.22 ± 4.42	3.17 ± 2.38	24.52 ± 176.90	0.252
Cholesterol (g)	225.48 ± 145.91	299.37 ± 190.77	281.99 ± 194.92	0.069
Simple sugar (g)	5.61 ± 11.85	6.27 ± 11.79	15.38 ± 32.65	0.008
Iron (mg)	9.19 ± 3.65	11.05 ± 8.40	12.29 ± 15.38	0.084
Zinc (mg)	7.29 ± 2.29	33.44 ± 209.67	7.78 ± 2.73	0.981
Vitamin E (mg)	5.25 ± 3.33	6.17 ± 6.81	9.03 ± 16.16	0.035
Vitamin B12 (μg)	3.07 ± 2.69	3.92 ± 3.80	3.63 ± 4.72	0.402
Folic acid (μg)	71.84 ± 105.50	146.05 ± 271.31	151.83 ± 140.16	0.014
Vitamin C (mg)	122.49 ± 79.81	125.64 ± 103.82	110.52 ± 88.71	0.450
EPA (mg)	51.14 ± 142.07	52.02 ± 81.32	77.29 ± 111.15	0.189
DHA (mg)	215.95 ± 887.38	82.12 ± 143.14	211.68 ± 550.07	0.968
Fasting glucose (mg/dL)	102.01 ± 15.77	101.68 ± 18.77	93.09 ± 10.56	0.001
HbA1c (%)	5.79 ± 0.61	5.68 ± 0.61	5.47 ± 0.46	0.001

^1^ Tertiles of dietary pattern scores; ^2^ *p* trend was calculated using generalized linear models for continuous variables. Abbreviations: BMI, body mass index. EPA, eicosapentaenoic acid. DHA, docosahexaenoic acid. HbA1c, glycated hemoglobin.

**Table 7:** Risk of IFG by the tertiles of dietary patterns under multivariate models.

Dietary Patterns		Crude Model	Multivariate Model 1 ^1^	Multivariate Model 2 ^2^
Western	T1 ^3^	1.00	1.00	1.00
	T2	3.11 (1.47 to 6.58)	3.05 (1.37 to 6.76)	2.95 (1.33 to 6.57)
	T3	3.61 (1.70 to 7.66)	3.39 (1.53 to 7.52)	3.30 (1.48 to 7.35)
	*p* trend ^4^	0.001	0.003	0.004
Prudent	T1 ^3^	1.00	1.00	1.00
	T2	1.18 (0.59 to 2.36)	1.12 (0.53 to 2.36)	1.17 (0.55 to 2.48)
	T3	1.21 (0.60 to 2.43)	1.02 (0.48 to 2.19)	1.11 (0.51 to 2.41)
	*p* trend ^4^	0.595	0.954	0.794
Convenience	T1 ^3^	1.00	1.00	1.00
	T2	1.05 (0.51 to 2.14)	0.89 (0.41 to 1.92)	0.89 (0.41 to 1.94)
	T3	2.40 (1.19 to 4.86)	2.04 (0.95 to 4.38)	2.01 (0.93 to 4.33)
	*p* trend ^4^	0.014	0.063	0.071
Asian traditional	T1 ^3^	1.00	1.00	1.00
	T2	1.68 (0.83 to 3.37)	1.95 (0.92 to 4.16)	1.85 (0.86 to 3.97)
	T3	1.51 (0.74 to 3.08)	1.44 (0.67 to 3.09)	1.38 (0.64 to 2.97)
	*p* trend ^4^	0.255	0.365	0.430
Continental	T1 ^3^	1.00	1.00	1.00
	T2	0.91 (0.46 to 1.80)	0.87 (0.42 to 1.83)	0.85 (0.40 to 1.78)
	T3	0.36 (0.17 to 0.74)	0.44 (0.20 to 0.97)	0.42 (0.19 to 0.92)
	*p* trend ^4^	0.006	0.044	0.032

^1^ Adjusted for age and body mass index; ^2^ Model 1 with additional adjustment for total energy intake; ^3^ Tertiles of dietary pattern scores; ^4^ Tests for trend were conducted by assigning the median value to each tertile of food intake as a continuous variable. Abbreviations: IFG, impaired fasting glucose.

**Table 8:** Risk of abnormal HbA1c by the tertiles of dietary patterns under multivariate models.

Dietary Patterns		Crude Model	Multivariate Model 1 ^1^	Multivariate Model 2 ^2^
Western	T1 ^3^	1.00	1.00	1.00
	T2	1.59 (0.80 to 3.18)	1.57 (0.75 to 3.32)	1.54 (0.73 to 3.25)
	T3	1.74 (0.87 to 3.48)	1.65 (0.78 to 3.47)	1.61 (0.76 to 3.41)
	*p* trend ^4^	0.117	0.192	0.214
Prudent	T1 ^3^	1.00	1.00	1.00
	T2	1.59 (0.80 to 3.18)	1.50 (0.71 to 3.14)	1.55 (0.73 to 3.26)
	T3	1.74 (0.87 to 3.48)	1.42 (0.67 to 3.01)	1.50 (0.70 to 3.21)
	*p* trend ^4^	0.117	0.367	0.301
Convenience	T1 ^3^	1.00	1.00	1.00
	T2	0.81 (0.40 to 1.62)	0.67 (0.32 to 1.42)	0.66 (0.31 to 1.41)
	T3	1.96 (0.98 to 3.92)	1.45 (0.69 to 3.07)	1.42 (0.67 to 3.02)
	*p* trend ^4^	0.056	0.327	0.351
Asian traditional	T1 ^3^	1.00	1.00	1.00
	T2	1.37 (0.69 to 2.72)	1.58 (0.75 to 3.31)	1.52 (0.72 to 3.21)
	T3	1.69 (0.84 to 3.38)	1.70 (0.80 to 3.61)	1.66 (0.78 to 3.52)
	*p* trend ^4^	0.139	0.166	0.189
Continental	T1 ^3^	1.00	1.00	1.00
	T2	0.76 (0.38 to 1.51)	0.81 (0.39 to 1.71)	0.79 (0.38 to 1.67)
	T3	0.29 (0.14 to 0.60)	0.38 (0.18 to 0.82)	0.37 (0.17 to 0.79)
	*p* trend ^4^	0.001	0.014	0.011

^1^ Adjusted for age and body mass index; ^2^ Model 1 with additional adjustment for total energy intake; ^3^ Tertiles of dietary pattern scores; ^4^ Tests for trend were conducted by assigning the median value to each tertile of food intake as a continuous variable. Abbreviations: HbA1c, glycated hemoglobin.
